# Electrophysiological Characteristics in Depressive Personality Disorder: An Event-Related Potential Study

**DOI:** 10.3389/fpsyg.2018.02711

**Published:** 2019-01-10

**Authors:** Hong-Hua Yu, Si-meng Gu, Fang-Min Yao, Zhi-Ren Wang, Wen-Qing Fu

**Affiliations:** ^1^Beijing Huilongguan Hospital, Peking University, Beijing, China; ^2^School of Psychology, Jiangsu University Medical Center, Zhenjiang, China; ^3^Department of Psychology, Medical College of Suzhou University, Suzhou, China

**Keywords:** depressive personality disorder, event related potential, N350, emotion, word classification

## Abstract

This study aimed to investigate the neurophysiological characteristics of young people with depressive personality disorder using event-related potentials (ERP). To explore the effects of visual-emotional words on ERP, mainly N350, we recruited 19 individuals with a depressive personality disorder and 10 healthy controls. ERP were recorded while the subjects took decisions on target words that were classified into three categories: emotionally positive, negative, and neutral. The ERP signals were then separately averaged according to the subjects’ classifications. Data analysis showed that the amplitude of N350 was larger in response to positive and negative words than to neutral words. The latency of N350 was longer in negative words, in contrast with positive and neutral words. However, no difference was found between the two groups. These results suggest that neurophysiological characteristics of young people with a depressive personality disorder in visual-emotional word processing have not yet been influenced by their personality traits. To some extent, N350 reflected semantic processes and was not sensitive to participants’ mood state.

## Introduction

A great deal of evidence has accumulated in clinical neurophysiology concerning cognitive functions in depressive patients (Mao et al., 2005; Krompinger and Simons, 2011; Dai and Feng, 2012; Zhao et al., 2015; Kiang et al., 2017; Xie et al., 2018). However, only a few studies have focused on the depressive personality disorder (DPS) in healthy young populations (Shimizu et al., 2006). College is an important period for individuals in their lifelong psychological development. Childhood negative or traumatic experiences, the pressure of adapting to new environments, together with the uncertainties of the future, exert many negative effects on the personality composition of college students, some of whom develop depressive personality. Therefore, the purpose of the present study is to explore the neurophysiological characteristics of young people with depressive personality disorder.

Similar to clinically depressed subjects, individuals with depressive personality traits are usually somber, restrained, and socially regressive (Noordhof et al., 2018). Since the application of event-related potentials (ERP) technology has been undertaken to explore neural physiological aspects of depression (MacNamara et al., 2016; Kiang et al., 2017), many ERP abnormalities have been reported to be related to depression. For example, it was found that depressive patients had smaller N400s than controls, specifically for negative adjectives, suggesting that depression is associated with stronger-than-normal functional neural links between self-concept and negative characteristics (Kiang et al., 2017). A study aimed to investigate the intensity of evaluation of social stimuli in depression and showed that participants with depression had higher intensity scores for sad faces compared with the normal control group, longer reaction times for all faces compared with other groups, and higher P1 and P2 amplitude for sad faces compared with other faces. This finding suggested that the participants with depression were more receptive to negative facial expressions (Dai and Feng, 2012). MacNamara examined emotional processing abnormalities among 97 outpatients with generalized anxiety disorder (GAD) or major depressive disorder (MDD) using the late positive potential (LPP) and found that both diagnoses were associated with increased LPP. Both MDD and GAD were associated with an increased reaction time to targets that followed emotional pictures (MacNamara et al., 2016). Hui Xie examined the intentional forgetting of negative and neutral material in individuals with depressive tendencies. The results indicated that individuals with depressive tendencies had difficulties suppressing the memory encoding with negative words, while the suppression of memory encoding of neutral words was relatively intact. Furthermore, compared to individuals without depressive tendencies, depressive individuals had larger word-evoked P2 and late positive potential for negative items, as well as enhanced cue-evoked P1 and N2 for the negative items which were required to be forgotten (Xie et al., 2018).

According to the previous studies, N350 was identified as a phonological/lexical component (Bentin et al., 1999; Spironelli and Angrilli, 2009; Spironelli and Angrilli, 2015); moreover, other researchers found that negative components during 300–400 ms activated by emotional words significantly differed from those by neutral words (Kissler et al., 2006; Kiefer et al., 2007; Herbert et al., 2008; Fan et al., 2016). These results put forward a question concerning why there were differences in these ERP components between emotional and neutral words if they only manifested phonological/lexical processing. As we know, phonological units of words are associated with meanings; yet we can recognize phonological units as words without knowing exactly what they mean. Therefore, these 300–400 ms negative components should participate in at least part of the semantic task in visual word recognition. There was evidence for N400 to be sensitive to semantic deviations for stimuli with a semantic context (Kiefer, 2002; Briesemeister et al., 2014). However, to the best of our knowledge, studies exploring whether N350 will be affected by emotional content of words are rare. One of the goals of the present study was to bridge this gap.

Another reason why we examined N350 was that a number of studies suggested that both a word’s emotional content and the participants’ emotional state may affect the N400 ERP response (Chung et al., 1996; Federmeier et al., 2001; Kiefer, 2002). Yet, for the time being, very few studies have investigated the impact of the subjects’ emotional state on the N350 component.

In the present study, 19 undergraduate students who met the diagnostic criteria of depressive personality disorder (DPS) and 10 healthy undergraduate students participated. Our goals included examining how visual-emotional words affected word recognition processing in a mild depressive state of young persons through recorded ERP, mainly the N350 component, and whether the emotional state of participants would have any effect on the ERP or N350.

## Materials and Methods

### Subjects

Participants were drawn from an initial sample of 1999 undergraduate students from Grade One and Grade Two of Suzhou University. Fifty-three students with depressive personality traits screened by Personality Disorder Diagnosis Questionnaire, Fourth Edition (PDQ^4+^) were further diagnosed by the Personality Disorder Interview (PDI-IV) semi-structured interview. Nineteen individuals finally diagnosed with depressive personality disorder took park in the study as the DPS group: (9 female, mean age 20.21 years). All 19 subjects did not take any antidepressants or other psychotropic drugs. Ten healthy undergraduates participated as the normal control group (6 female, mean age 19.70 years). With respect to exclusion criteria, participants in both groups should not have been diagnosed with psychiatric disorders including schizophrenia, affective disorder, bipolar disorder, intellectual disability, substance abuse, and dementia, as well as chronic medical disorders including endocrine disorders, cardiovascular disorders, and diseases related to the central nervous system. This study protocol conformed to the ethical guidelines of Suzhou University and was approved by the institutional ethics committee (the Ethic Committee of Suzhou University). Written informed consent was obtained from each participant.

### Questionnaires

#### Personality Disorder Diagnosis Questionnaire, Fourth Edition (PDQ^+4^)

The PDQ^+4^, designed by Dr. Hyler according to the Diagnostic and Statistical Manual of Mental Disorders Fourth Edition (DSM-IV) diagnostic criteria for personality disorders, was used to assess whether an individual has personality disorders traits. Dr. Jian Yang translated it into Chinese in 1996. The validity and reliability of the questionnaire were tested by Yun-ping Yang in 2002. Research showed that the PDQ^+4^ is high in sensitivity and low in specificity and could be used as a personality disorder screening questionnaire (Yang et al., 2002).

#### Personality Disorder Interview (PDI-IV)

The PDI-IV, a semi-structured interview instrument, developed by Dr. Thomas A. Widiger according to the DSM-IV, was employed to diagnose the 12 personality disorders. The PDI-IV provided a set of 3–5 consistent questions for each diagnostic criterion of the 12 personality disorders. The interviewer can give more detailed explanations for each question to ensure that the subjects understand what the questions mean. The PDI-IV evaluation is in the form of a 3-point scale, that is, from 0 to 2, where “0” means that there is no corresponding symptom, “1” is equal to the existence of the corresponding symptom, “2” is equal to the existence of a more serious or more corresponding symptoms. The original scale of the positive demarcation was 4–5 points, and the test takes about 20–30 min. The reliability between different interviewers was 0.34–0.89, with the mean being 0.74 (Yang et al., 2000).

### Experimental Instruments and Stimuli

Electroencephalographs (EEGs) were recorded from 30 scalp locations over both hemispheres by a 64-channel electroencephalograph using 64 electrodes. The general experimental procedure, selection of stimuli, and stimulus presentation closely followed that of former studies (Blackburn et al., 1990; Dietrich et al., 2000; Yao et al., 2004). Two hundred Chinese mood words (each word containing two Chinese characters) were selected from the standardized Chinese mood words system, 40 of which were positive adjectives (i.e., industrious, modest, and dignified) as target stimuli accounting for 20% of the stimuli; 40 were negative adjectives (i.e., sad, gloomy, and angry) as target stimuli accounting for 20% of the stimuli; and 120 were neutral nouns (i.e., river, wood, and glass) as non-target stimuli accounting for 60% of the stimuli.

### Procedure

#### Research Team Members Training

The team consisted of two clinical psychologists and two graduate students majoring in medical psychology. The one-week training included studying the instruments, and practice for the interview was held before the use of the PDQ^+4^ and PDI-IV to ensure consistency in comprehension and practice between the team members. The PDQ^+4^ was distributed among the undergraduate students and the positively screen individuals were interviewed using the PDI-IV to diagnose the personality disorders.

#### Stimuli and Tasks

The subjects were 19 undergraduate students, who met the depressive personality disorder diagnostic criteria and 10 normal students. Both groups were instructed to perform a target words classification task. The experimental procedure was programmed using E-prime. The 200 Chinese words were presented randomly on the computer screen. Each word was presented for 500 ms, with the SOA (stimulus onset asynchrony) being 1800 ms. The whole experiment consisted of 6 blocks. Block 1 was presented as a sample trial; Blocks 2–6 were then presented subsequently, each of which lasted 4 min. The subjects were instructed to stay awake with their eyes-opened and were tested in a quiet room (sound was attenuated and the temperature was controlled at 25°C). They were instructed to respond by pressing keys when they saw mood adjectives. In this experiment, odd-numbered subjects were requested to press the F key rapidly with the left forefinger as soon as they saw positive mood words, to press the J key with the right forefinger as soon as they saw negative words, and not to press any key when they saw neutral words. Even-numbered subjects were requested to press the opposite pattern of keys. The reaction time and accuracy of the subjects’ words classification were recorded.

#### ERP Recording and Quantification

Electroencephalograms were recorded using bilateral mastoids as reference. Horizontal electro-oculograms (EOGs) were recorded differentially from the outer canthi of each eye and vertical EOGs from supra and infraorbital sites. Electrode cream was applied between the electrodes and scalp to maintain the impedances at 5k℧ or less. EEGs were recorded continuously and processed off-line overlaid. EOGs were corrected automatically using Neuroscan. Other artifacts were also fully rejected. Recorded EEGs were classified into three kinds of ERPs as positive, negative, and neutral words, and overlaid separately; the amplitude and peak latency were measured automatically. N350 was investigated in a time-window of 300–400 ms and was calculated as the mean amplitude (Bentin et al., 1999; Schendan and Kutas, 2007). We defined interest regions of Fz, F3, F4, Cz, and Pz to test the N350 component (Shen et al., 2013), and the average of the regions was calculated. The scalp distribution was presented in topography.

### Statistics

A statistical analysis was conducted with *t*-test and chi-square for behavioral data. EEG data were analyzed with repeated measurement ANOVA, which included between-subjects factor of group (two levels: DPS and normal control) and two within-subjects factors: word types (three levels: positive, negative, and neutral) and electrode site (five levels: Fz, F3, F4, Cz, and Pz).

## Results

### Comparison of the Two Groups in Word Classification

A *t*-test showed that the average response time in the DPS group (727.70 ± 327.63) was significantly prolonged as compared to that in the normal control group (681.53 ± 360.33) (*t* = 3.077, *p* < 0.01). A chi-square test showed that the average accuracy in the two groups was not significantly different (χ2 = 0.514, *p* > 0.05).

### ERPs Evoked by Mood Words in DPS and Control Groups

**FIGURE 1:**
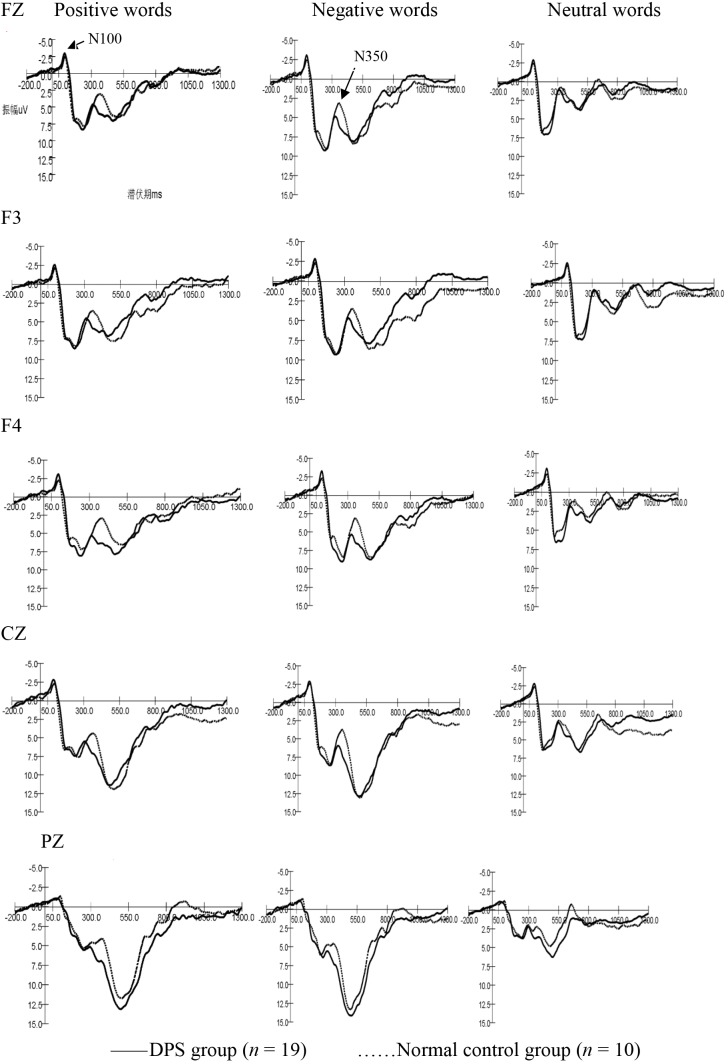
The ERP waveforms evoked by three kinds of words in normal controls and the DPS group.

Negative ERPs evoked by mood words in the two groups were named as N100 and N350 by latency period and components’ serial number (Figure 1).

### N350 Amplitude Analysis

**FIGURE 2:**
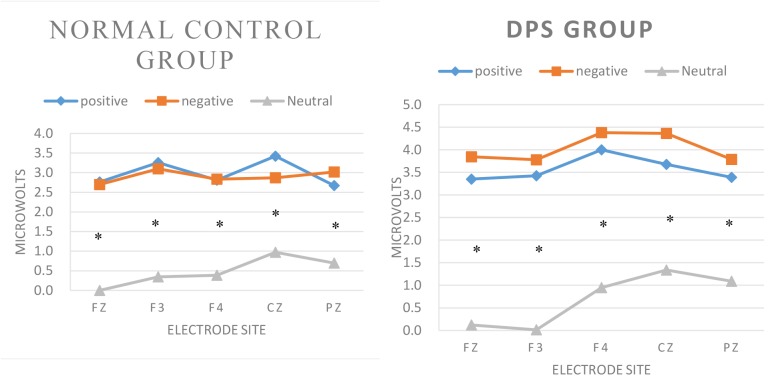
Amplitude analysis: Significant three-way group by word type by electrode site interaction during word classification tasks. Asterisks: significant pairwise *t*-tests.

Repeated measurement ANOVA computed on N350 amplitude showed a significant main effect of word type: *F*(2, 26) = 29.599, *p* < 0.001. Pairwise *t*-tests revealed larger N350 amplitude (i.e., more negative) for positive and negative words as compared to neutral words (*p* < 0.001). There were smaller N350 amplitudes for positive words as compared to negative words, but the difference was not significant. Concerning interactions, the three-way group by word type by electrode site interaction revealed no interactions between group, electrode site, and word type: *F*(8, 20) = 0.604, *p* > 0.05. The result is illustrated in Figure 2.

### N350 Latency Analysis

**FIGURE 3:**
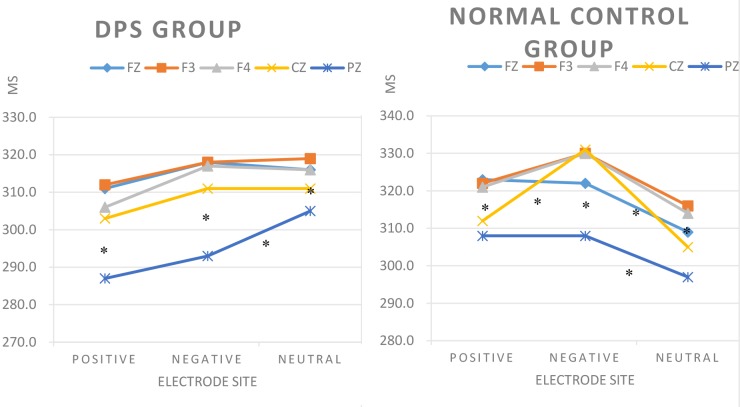
Latency analysis: Significant three-way group by word type by electrode site interaction during word classification tasks. Asterisks: significant pairwise *t*-tests.

Repeated measurement ANOVA computed on N350 latency showed significant main effects electrode site: *F*(2, 26) = 4.485, *p* < 0.01 and word type: *F* = 3.603, *p* < 0.05. The follow up pairwise *t*-tests revealed that Pz had shorter N350 latency than any other electrodes (*p* < 0.01). Negative words had longer latency than positive words (*p* < 0.05). Positive and neutral words, as well as negative and neutral words, did not differ. There were no interactions between the three factors: *F*(8, 20) = 0.735, *p* > 0.05. The pairwise comparisons are illustrated in Figure 3.

### Brain Topography of ERP Components

**FIGURE 4:**
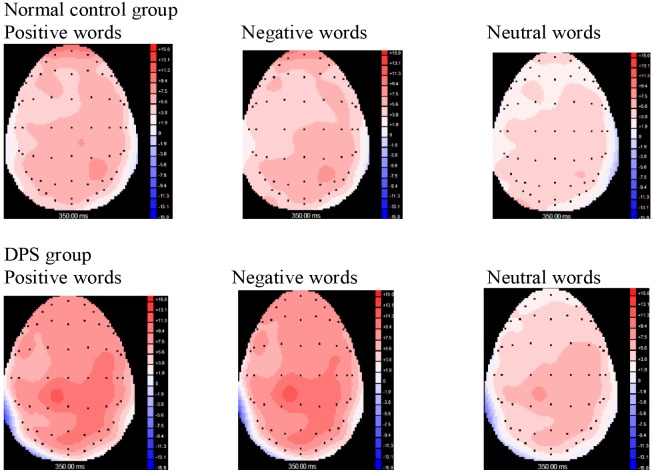
Brain topography of ERPs (N350) evoked by word processing of the two groups.

The most obvious amplitude changes of N350 evoked by positive and negative words in normal controls were located in the frontal region (Fz), while those evoked by neutral words had no prominent changes (Figure 4). The obvious amplitude changes of N350 evoked by positive and negative words in the DPS group were mainly distributed in the right parietal region (P4) and the right occipital region (O3), and that evoked by neutral words had a similar distribution, but less in extent compared to normal controls.

## Discussion

The aim of this study was to investigate the influence of the emotional content of words on brain word recognition processing in non-medicated college students with a depressive personality disorder (*n* = 19) compared with a control group (*n* = 10) utilizing event-related brain potentials (ERPs). In a continuous word recognition paradigm, subjects were instructed to discriminate words that were classified into three different categories of emotional content (positive, negative, and neutral).

Behavioral data showed that the response time was longer in the DPS group than in the normal control group. This result was consistent with several earlier reports (Mao et al., 2005; Shimizu et al., 2006; MacNamara et al., 2016). There was evidence that even a mild depressive state might lead to cognitive impairment such as the inability to concentrate on a task, having difficulties in comprehensive reading, and slow response to stimuli (Rosenberg et al., 2013; Chan et al., 2018). Hence, the inefficient response performance might be due to cognitive impairment in depressive individuals.

The negative deflection around 400 ms after word presentation has been known to contribute to semantic processing in healthy individuals (Kiefer et al., 2007; Herbert et al., 2008). Previous studies reported that pathological responses to words occur at a similar latency after word presentation in depressive subjects. Hideki Shimizu investigated the relationship between the ERP response of emotive words and the depression score in 35 healthy subjects with both high and low Beck Depression Inventory scores and found that the high score group had a more enhanced N400 than that in the low score group. Moreover, the peak latency of the high-scoring group was significantly longer compared to the low-scoring group at P3, Cz, and Pz sites, while there was no difference in amplitude between positive and negative words (Shimizu et al., 2006). Another study found that patients with depression had smaller N400s than controls, specifically for negative adjectives (Kiang et al., 2017). In the present study, there was no difference in N350 between the DPS and normal control groups. As we know, several researchers found that N400 was sensitive to the participants’ mood state in visual word processing (Chung et al., 1996; Federmeier et al., 2001; Kiefer, 2002). Our findings might suggest that the individual’s mood state cannot be reflected as early as 350 ms after the stimuli onset.

Although there was no difference between the two groups, the amplitude of N350 activated by emotional words was significantly larger than neutral words, while there was no significant difference between positive and negative words. According to the previous studies, N350 was identified as a phonological/lexical component (Bentin et al., 1999; Spironelli and Angrilli, 2009, 2015). However, the contradiction was that if N350 only reflected the phonological process, minor differences should exist between emotional and neutral words because we can recognize phonological units as words without knowing exactly what they mean. Furthermore, different from letter-words in Latin languages, Chinese characters are pictographs, which imply little phonology. Therefore, this result implied that the neural system had already started the semantic processing at the peak latency of about 350 ms after stimulus onset.

This hypothesis was supported by other researchers. Johanna Kissler concluded in a review article concerning visual-emotional word processing with ERP that emotional word content can activate word processing at all stages from access to word meaning (around 200 ms), to contextual integration (around 400 ms), evaluation, and memory encoding (around 600 ms). Of importance is the fact that the interpretation of N400 has changed from an index of semantic access to a signature of the interaction between single-word semantics and context. Accordingly, an abundance of evidence demonstrates that some aspects of word meaning must be active before N400 is elicited (Kissler et al., 2006). The present study adds new evidence to this supposition.

Regarding how the amplitude of N350 is larger for emotional words than neutral words, one of the most plausible explanations is that a larger amplitude reflects more neurons activated in emotional word processing. Subcortical structures, most prominently the amygdala, have been implied in all stages of the emotional content-driven amplification process (Naccache et al., 2005; Almeida et al., 2014). An fMRI study found that both negative and positive words activated the amygdala, and negative word processing revealed a positive correlation between amygdala activity and scores of trait anxiety and subclinical depression. During negative versus neutral word reading, subjects with high trait anxiety also showed a stronger functional coupling between the left amygdala and left dorsolateral prefrontal cortex (DLPFC) (Laeger et al., 2012). Therefore, subcortical, primarily amygdala, activity may be a source of cortical-amplifying mechanisms in response to emotional stimuli visible in ERPs.

An N350 latency analysis showed that negative words had longer latency than positive words. Again, no significant difference was found between the two groups. So far, we have not found the N350 component reported in depressive subjects during word recognition. In Hideki Shimizu’s study, for the N400 component in the positive/negative series, the peak latency of the high-scoring group was significantly longer compared to the low-scoring group at P3, P4, Cz, and Pz sites. Hideki considered that the large and delayed N400 component was due to the enhancement and continuation of semantic processing of emotive words in high-scoring subjects, relative to that in the low-scoring group. However, there were contrary results regarding the latency of ERP. Jiu Chen used a visual-emotional oddball paradigm to manipulate the processing of emotional information, while ERP was recorded in patients with major depression, and found that patients with recurrent major depression had longer N170 latencies when identifying happy and neutral faces, but shorter N170 latencies when identifying sad faces. With respect to this result, Jiu Chen suggested it might due to a negative bias of patients with recurrent major depression who were more aroused by sad faces than other emotions(Chen et al., 2014). The presumption for these controversial results was that participants in this and Hideki Shimizu’s studies were all relatively healthy young people who had depressive personality traits instead of being patients with major depression. This might suggest that the persons with a depressive personality had not yet developed a negative bias, which plays an important role in the maintenance of depressive symptoms. Their longer latency to negative words might reflect the natural bias toward positive information in normal persons. There is evidence supporting this explanation. Herbert investigated the extent to which emotional connotation influences cortical potentials during reading and revealed that healthy subjects may have a natural bias toward pleasant information, facilitating late ERPs (N400, LPP) to pleasant adjectives as well as their superior recall (Herbert et al., 2008).

The lack of group differences in the current study probably lay on two reasons. First, the sample size of normal controls was relatively small, which could affect the explanation of our results. Second, all participants were college students whose social function was normal, though the DPS subjects met the diagnostic criteria of depressive personality disorder.

## Conclusion

In sum, the current study demonstrated the effects of emotional word content on N350 cortical indices during a continuous word recognition paradigm in DPS and normal control groups. For the N350 component, our analysis was exploratory because, as yet, very few studies have investigated the impact of emotional content on this component. However, the effects of facilitated cortical processing in emotional words, reflected by larger N350 amplitudes for positive and negative words as opposed to neutral words and longer N350 latency for negative words compared to positive and neutral words, provide strong evidence for the notion that N350 also reflected semantic processes and was not sensitive to participants’ mood state. No difference between the two groups might imply that neurophysiological characteristics of young people with a depressive personality disorder have not yet been influenced by their personality traits.

## Author Contributions

W-QF and Z-RW designed the experiments. H-HY, F-MY, and S-mG performed the experiments. H-HY and S-mG wrote the manuscript.

## Conflict of Interest Statement

The authors declare that the research was conducted in the absence of any commercial or financial relationships that could be construed as a potential conflict of interest.
